# Influence of Genetic Polymorphisms of Tumor Necrosis Factor Alpha and Interleukin 10 Genes on the Risk of Liver Cirrhosis in HIV-HCV Coinfected Patients

**DOI:** 10.1371/journal.pone.0066619

**Published:** 2013-06-26

**Authors:** Sara Corchado, Mercedes Márquez, Montserrat Montes de Oca, Paula Romero-Cores, Clotilde Fernández-Gutiérrez, José-Antonio Girón-González

**Affiliations:** 1 Unidad de Enfermedades Infecciosas, Hospital Universitario Puerta del Mar, Cádiz, Spain; 2 Servicio de Microbiología, Hospital Universitario Puerta del Mar, Cádiz, Spain; Pohang University of Science and Technology, Republic of Korea

## Abstract

**Objective:**

Analysis of the contribution of genetic (single nucleotide polymorphisms (SNP) at position -238 and -308 of the tumor necrosis factor alpha (TNF-α) and -592 of the interleukin-10 (IL-10) promotor genes) and of classical factors (age, alcohol, immunodepression, antirretroviral therapy) on the risk of liver cirrhosis in human immunodeficiency (HIV)-hepatitis C (HCV) virus coinfected patients.

**Patients and Methods:**

Ninety one HIV-HCV coinfected patients (50 of them with chronic hepatitis and 41 with liver cirrhosis) and 55 healthy controls were studied. Demographic, risk factors for the HIV-HCV infection, HIV-related (CD4+ T cell count, antiretroviral therapy, HIV viral load) and HCV-related (serum ALT concentration, HCV viral load, HCV genotype) characteristics and polymorphisms at position -238 and -308 of the tumor necrosis factor alfa (TNF- α) and -592 of the interleukin-10 (IL-10) promotor genes were studied.

**Results:**

Evolution time of the infection was 21 years in both patients’ groups (chronic hepatitis and liver cirrhosis). The group of patients with liver cirrhosis shows a lower CD4+ T cell count at the inclusion in the study (but not at diagnosis of HIV infection), a higher percentage of individuals with previous alcohol abuse, and a higher proportion of patients with the genotype GG at position -238 of the TNF-α promotor gene; polymorphism at -592 of the IL-10 promotor gene approaches to statistical significance. Serum concentrations of profibrogenic transforming growth factor beta1 were significantly higher in healthy controls with genotype GG at -238 TNF-α promotor gene. The linear regression analysis demonstrates that the genotype GG at -238 TNF-α promotor gene was the independent factor associated to liver cirrhosis.

**Conclusion:**

It is stressed the importance of immunogenetic factors (TNF-α polymorphism at -238 position), above other factors previously accepted (age, gender, alcohol, immunodepression), on the evolution to liver cirrhosis among HIV-infected patients with established chronic HCV infections.

## Introduction

Chronic infection with hepatitis C virus (HCV) is characterized by a broad spectrum of clinical manifestations that can culminate in decompensated cirrhosis. An estimated 20–30% of infected individuals will develop cirrhosis while others largely remain asymptomatic [1].

Liver fibrosis is the most important prognostic factor in chronic HCV-infected patients [2]. The hepatic stellate cell is the major cell responsible for fibrosis in the liver, with activation of these cells being a key fibrotic event [3], [4]. The influence of inflammatory mediators in this liver process has been theorized [5]: impaired intestinal permeability and microbial translocation favour the presence of increased serum endotoxin or lipopolysaccharide (LPS) concentration in patients with chronic hepatopathies [6]. After been recognised by a toll-like receptor (toll-like receptor 4 –TLR4-), endotoxin signalling triggers a cascade that leads to proinflammatory cytokine production, including tumour necrosis factor (TNF)-α synthesis [7], [8]. TLR4 can also detect endogenous ligands, many of which are abundant during tissue injury, such as hyaluronan, fibronectin and heat shock proteins [7].

TNF-α can potentially affect liver fibrogenesis by stimulating hepatic stellate cells [9]. The pathogenic importance of TNF-α in liver disease has been previously demonstrated: besides the increased concentration of TNF-α in the liver of patients with chronic hepatitis C [10], it has been observed that serum levels of this cytokine are correlated with histological grading score of hepatitis [11]; moreover, patients with increased serum levels of TNF-α or their receptors showed a reduced survival [12].

A wide range of TNF-α production has been observed and can be attributed to polymorphisms in the TNF-α promoter and their corresponding extended HLA haplotypes [13]. In particular, two common biallelic variants at the -308 (G or A) and -238 (G or A) positions of the TNF-α promoter have been the first to receive attention [14]. The TNF-α polymorphism in -308 and -238 positions of the TNF promoter has been involved in the variability of the histological severity of chronic hepatitis C infection [15], [16], [17], [18], [19]. A possible explanation to the variable progression of liver fibrosis was provided by Wilson et al [20] with the demonstration that carriage of the -308 allele A, a much stronger transcriptional activator than -308 allele G in reporter gene assays, has direct effects on TNF-α gene regulation which may be responsible for the association with higher constitutive and inducible levels of TNF-α. However, a metaanalysis of 11 different studies about this topic has not detected association between this polymorphism and the risk of liver cirrhosis [21]. The -238 allele A functional consequences are not yet clear compared with -238 allele G [22].

Other cellular cytokine genes in which genetic variation has been examined within the context of fibrotic disease include interleukin-10 (IL-10). IL-10 is an anti-inflammatory cytokine that down regulates the synthesis of pro-inflammatory cytokines, including TNF-α, and has a modulatory effect on hepatic fibrogenesis [15]. IL-10 levels differ widely between individuals, possibly because of polymorphisms in the promoter region of the IL-10 gene [23]. IL-10 polymorphisms have been studied in the context of hepatic fibrosis, with controversial results [24], [25], [26], [27], [28].

Additionally to the possible contribution of genetic factors, evolution to cirrhosis in HCV-induced chronic hepatitis is dependent of the presence of coinfection by hepatitis B virus, alcohol ingestion, or immunodeficiency (immunomodulation to prevent graft rejection or HIV coinfection), among others [29]. Effectively, liver fibrosis progression to cirrhosis is faster in HIV-HCV coinfected individuals than in HCV-mono-infected subjects [30], [31]. In fact, complications of the hepatitis C virus (HCV) infection are one of the main causes of death in human immunodeficiency virus (HIV)-coinfected patients [32]. Thus, we would like to address the question of whether the possible genetic contribution to liver fibrosis progression might be overruled by more vigorous stimuli, such as the HIV coinfection. With this objective, we have assessed the influence of TNF-α and IL-10 single nucleotide polymorphisms on the progression of HCV-induced chronic hepatitis to cirrhosis in HIV-coinfected patients. Likewise, an analysis of serum concentration of molecules related with pro-inflammatory (TNF-α, interleukin-6 –IL-6-), anti-inflammatory (IL-10) and fibrogenic molecules (transforming growth factor beta 1 –TGF-β1-) was performed in healthy controls and patients with the diverse genotypes.

## Materials and Methods

### Design

This was a cross-sectional population association study.

### Patients

All subjects were consecutively recruited from a prospectively collected cohort of HIV-infected patients treated at the HIV outpatients' clinics of an university hospital. Caucasian patients with chronic HIV and HCV infection were studied. Patients were classified in those with cirrhosis (n = 41) and those with chronic hepatitis (n =  50). For the control group we studied a sample of healthy subjects recruited from voluntary hospital workers (n = 55), whose age and gender were comparable with the patients.

All were 18–70 years old. All patients had serum negative for hepatitis B surface antigen and antinuclear, antismooth muscle antibody and antimitochondrial antibody. None had hemochromatosis gene-related iron overload as assessed by serum iron markers, biopsy, and genotyping where indicated. Serum *α*1 antitrypsin and ceruloplasmin levels were normal.

### Definitions

Positive serum antibody to HIV was required for the diagnosis of HIV infection. Patients were classified according the 1993 Centers for Disease Control and Prevention classification of HIV infection. Spanish Group for AIDS Study guideliness (www.gesida.es) were used to indicate the antiretroviral treatment (HAART). Plasma HIV RNA load was quantified by polymerase chain reaction (PCR) assay; a value lower than 50 copies/ml was considered as undetectable HIV load. CD4+ T cell counts were determined by standard flow cytometry; values obtained at diagnosis of HIV infection and at the time of inclusion were considered.

Positive serum antibody to HCV and persistent (more than 6 months) HCV RNA were required for the diagnosis of chronic HCV infection. Diagnosis of chronic hepatitis or cirrhosis was established according to histological criteria when liver biopsy was performed [33], or by transient elastography, performed according to a standardized technique by one trained operator (JAGG) (according to data validated in HIV-HCV coinfected patients using liver biopsy as reference, patients with a liver stiffness > 14,6 kPa were classified as individuals with cirrhosis [34].

Duration of hepatitis C infection was estimated using an interviewer-assisted questionnaire assessing risk factors for HCV infection. The earliest exposure was designated as the point of acquisition [35].

Alcohol abuse was considered when an ingestion higher than 50 g/day during at least 5 years occurred.

### Laboratory determinations

HIV-1 infection was diagnosed using an EIA (Abbott Laboratories, North Chicago, IL, USA) and confirmed by New Lav Blot I (Bio-Rad, Marnes La Coquette, France). Plasma HIV-1 viral load was determined by the Cobas Amplicor HIV Monitor (Roche Diagnostics, Basel, Switzerland); the cutoff for undetectable viral load was 50 copies/μl. Blood CD4+ T-cell count were determined by flow cytometry (FAC Scan, Becton Dickinson Immunocytometry Systems, San Jose, CA, USA).

Antibodies anti-HCV were detected by 3^rd^ generation ELISA (Abbott Diagnostics, Chicago, USA). Plasma HCV RNA was detected by quantitative PCR (Amplicor HCV Monitor test; Roche Diagnostics, Basel, Switzerland). HCV genotype was identified by line-probe assay (INNOLiPA HCV; Innogenetics, Ghent, Belgium).

### TNF-α and IL-10 polymorphisms

The -308 TNF-α polymorphism (rs1800629) consists of a G to A substitution at position -308 in the proximal promoter of the TNF-α gene. The -238 TNF-α polymorphism (rs361525) consists of a G to A substitution at position -238 in the proximal promoter of the TNF-α gene. The IL-10 polymorphism (rs1800872) consists of a C to A substitution at position -592 in the proximal promoter of the IL-10. Each polymorphism was genotyped by predesigned Taqman assays (Applied Biosystems, Foster City, CA, EEUU), following the instructions of the manufacturer’s.

### Intracellular expression and LPS-stimulated secretion of cytokines

Blood samples from healthy controls with genotype GG (n = 10) and with genotype GA (n = 10) at -238 of the TNF-promoter, collected in pyrogen-free heparinized tubes ((Biofreeze, Costar, EEUU), were taken at 8 am to minimize the inﬂuence of circadian rhythms.

PBMC were incubated with combinations of fluorescein- (FITC), phycoerythrin (PE) and peridinin chlorophyll protein (PerCP)-labelled monoclonal antibodies (Becton-Dickinson, San Jose, CA, EEUU) and analysis of the unstimulated intracellular expression of TNF-α, IL-6, TGF-β1 and IL-10 was performed. Proportions of monocyte-expressing each cytokine were determined in PBMC by two-colour flow cytometry in a FACScalibur cytometer using Cell Quest and Paint-A-Gate software (Becton-Dickinson, San Jose, CA, USA).

In a separate set of experiments, whole-blood samples were mixed 1:1 with RPMI 1640 (Gibco, Germany) and lipopolysaccharide –LPS- (Escherichia coli 0111:B4, Difco, Detroit, MI) was added to the final concentrations of 1000 ng/mL; cells were stimulated for 4h at 37°C under 5% CO2 atmosphere. In addition, unstimulated baseline samples were obtained to act as a control. Concentration of TNF-α, IL-6, TGF-β1 and IL-10 in the supernatant of the cultures, after 24 hours incubation, was assayed.

### Concentration of pro- and anti-inflammatory and of fibrogenic molecules

Serum and culture supernatants concentrations of TNF-α, IL-6 and IL-10 were analyzed using Milliplex MAP kit High Sensitivity Human Cytokine (Millipore, Billerica, MA, USA). Serum TGF-β1 levels were detected by Quantikine Human Immunoassay (R&D, Minneapolis, MN, USA).

### Statistical analysis

Descriptive data were expressed as the median (25–75 interquartile range –IQR-) or as absolute number (percentage). Qualitative variables, including genotype distribution, were compared by the chi-square test or Fisher's exact test when necessary. Quantitative variables were compared using the Mann-Whitney U test or ANOVA when necessary. The Pearson’s correlation coefficient analysed the association between quantitative variables. The independent predictive value of genotypes and of those other variables associated to liver cirrhosis in the bivariate analysis was analyzed by linear regression analysis. A *p* value <0.05 was considered significant. The statistical analysis was carried out using the SPSS 15.0 statistical software package (SPSS Inc., Chicago, IL, USA).

### Ethical aspects

This study was performed according to the Helsinki Declaration. The project was approved by the hospital ethical research committee. Informed consent was obtained from each participant.

## Results

Ninety one HIV-HCV coinfected patients and fifty five healthy controls were studied. Table 1 shows the genotype distribution and allele frequencies of the TNF-α -238 G to A, TNF-α -308 G to A and IL-10 -592 C to A gene promoter single nucleotide polymorphisms for the control group and the HIV-HCV coinfected patients. Observed genotypic frequencies approximated to those expected based on allele frequency calculations, and thus conformed to Hardy-Weinberg equilibrium.

**Table 1:** Single nucleotide polymorphism of TNF-α and IL-10 genes studied in healthy controls and patients with HIV-HCV coinfection.

Single nucleotide polymorphism			Healthy controls (n = 55)	HIV-HCV coinfected patients (n = 91)	p
TNF rs361525 (- 238)	Genotype frequency (n, %)	GG	50 (93)	74 (86)	0,367
		GA	4 (7)	10 (12)	
		AA	0 (0)	2 (2)	
	**Allelic frequency**	G	0,96	0,93	0,535
		A	0,04	0,07	
**TNF rs1800629 (- 308)**	**Genotype frequency (n, %)**	GG	43 (78)	70 (81)	0,527
		GA	12 (22)	15 (17)	
		AA	0 (0)	2 (2)	
	**Allelic frequency**	G	0,88	0,90	0,821
		A	0,12	0,10	
**IL10 rs1800872 (- 592)**	**Genotype frequency (n, %)**	CC	24 (47)	43 (49)	0,758
		CA	21 (41)	38 (43)	
		AA	6 (12)	7 (8)	
	**Allelic frequency**	C	0,69	0,70	1,000
		A	0,31	0,30	

Note: In Genotype frequency, “n” expresses the number of patients in which the analysis of each polymorphism was successful. By that, it could be no coincidence among the sum of genotype frequencies and the absolute number of patients.

### Bivariate comparisons between HIV-HCV patients with chronic hepatitis and cirrhosis

The two patients’ groups, with and without cirrhosis, were comparable in terms of age, gender, risk factors for the infection, HCV viral load and viral genotypes. The frequency of alcohol use was significantly higher in patients with cirrhosis. More importantly, estimated duration of HCV infection was comparable in patients with chronic hepatitis and those with cirrhosis (Table 2). Analysis of HIV-related immune characteristics demonstrated that the CD4 T cell count at inclusion in the study was significantly lower in patients with cirrhosis, although CD4 T cell at diagnosis of HIV infection showed no significant differences. The percentages of patients with undetectable HIV viral load due to antiretroviral therapy were similar in both groups. HCV-related characteristics demonstrated that there were no significant differences between serum ALT concentration, HCV genotype, and HCV viral load of both groups.

**Table 2:** Demographic, clinical, virological and immunological characteristics of HIV-HCV coinfected patients with chronic hepatitis or liver cirrhosis.

	HIV-HCV coinfected patients with chronic hepatitis (n = 50)	HIV-HCV coinfected patients with liver cirrhosis (n = 41)	p
**General characteristics**			
Age (years)	45 (40–49)	47 (44–49)	0,056
Male sex (n, %)	39 (78)	36 (88)	0,275
Drug use as risk factor (n, %)	30 (60)	27 (66)	0,665
Evolution of the infection (years)	21 (19–24)	22 (19–25)	0,716
Alcohol ingestion (> 50 g/day) (n, %)	23 (49)	32 (78)	0,008
**HIV-related characteristics**			
CD4+ T lymphocytes/mm3 at HIV diagnosis	188 (110–306)	146 (60–246)	0,116
CD4+ T lymphocytes/mm3 at inclusion	386 (276–574)	333 (156–392)	0,002
CDC C stage (n, %)	20 (44)	19 (49)	0,667
Patients in antiretroviral therapy (n, %)	47 (94)	39 (95)	1,000
Undetectable HIV viral load (<50 copies/ml) at inclusion	39 (78)	32 (78)	1,000
**HCV-related characteristics**			
Diagnosis by liver biopsy (n, %)	32 (64)	31 (76)	0,261
Liver stiffness (Transient elastometry) (kPa) (median, range)	8,4 (7,1 – 13,0)	28,4 (15,4 – 76,8)	<0,001
HCV genotype 1 or 4 (n, %)	34 (68)	23 (56)	0,342
HCV viral load (1000 x IU/l) at inclusion	1192 (206–3133)	875 (288–2570)	0,906
ALT (UI/l)	40 (31–56)	45 (33–85)	0,544

Different distributions were noted in the single nucleotide polymorphisms among the patient groups (Table 3). Specifically, the TNF-α -238 A alleles were significantly more common in patients who suffered from chronic hepatitis than in those patients with cirrhosis. Differences in the distribution of IL-10 genotypes approach to significance. These differences persisted when only those patients diagnosed by liver biopsy (chronic hepatitis, n = 32; liver cirrhosis, n = 31) were considered (data not shown).

**Table 3:** Single nucleotide polymorphism of TNF-α and IL-10 genes studied in patients with HIV-HCV coinfection with chronic hepatitis or liver cirrhosis.

Single nucleotide polymorphism			HIV-HCV coinfected patients with chronic hepatitis (n = 50)	HIV-HCV coinfected patients with liver cirrhosis (n = 41)	p
**TNF rs361525 (- 238)**	**Genotype frequency (n, %)**	GG	36 (77)	38 (97)	0,009
		GA	10 (21)	0 (0)	
		AA	1 (2)	1 (3)	
**TNF rs1800629 (- 308)**	**Genotype frequency (n, %)**	GG	39 (80)	31 (82)	0,939
		GA	9 (18)	6 (16)	
		AA	1 (2)	1 (3)	
**IL10 rs1800872 (- 592)**	**Genotype frequency (n, %)**	CC	29 (59)	14 (36)	0,072
		CA	16 (33)	22 (56)	
		AA	4 (8)	3 (8)	

Note: In Genotype frequency, “n” expresses the number of patients in which the analysis of each polymorphism was successful. By that, it could be no coincidence among the sum of genotype frequencies and the absolute number of patients.

Analyses of the association between epidemiological, clinical, HIV- or HCV-related characteristics and TNF-α -238 phenotypes according to polymorphisms did not shown any significant differences. In brief, age, sex, alcohol ingestion, CD4+ T cell count at diagnosis and at inclusion, percentage of patients with undetectable HIV load, HCV genotypes, HCV load or serum ALT concentration were similar in patients with genotype GG or GA/AA of TNF-α -238 (p > 0,05 in each case).

### Relation among the detected polymorphisms and the serum concentrations of pro- and anti-inflammatory and profibrogenic molecules

**Figure 1:**
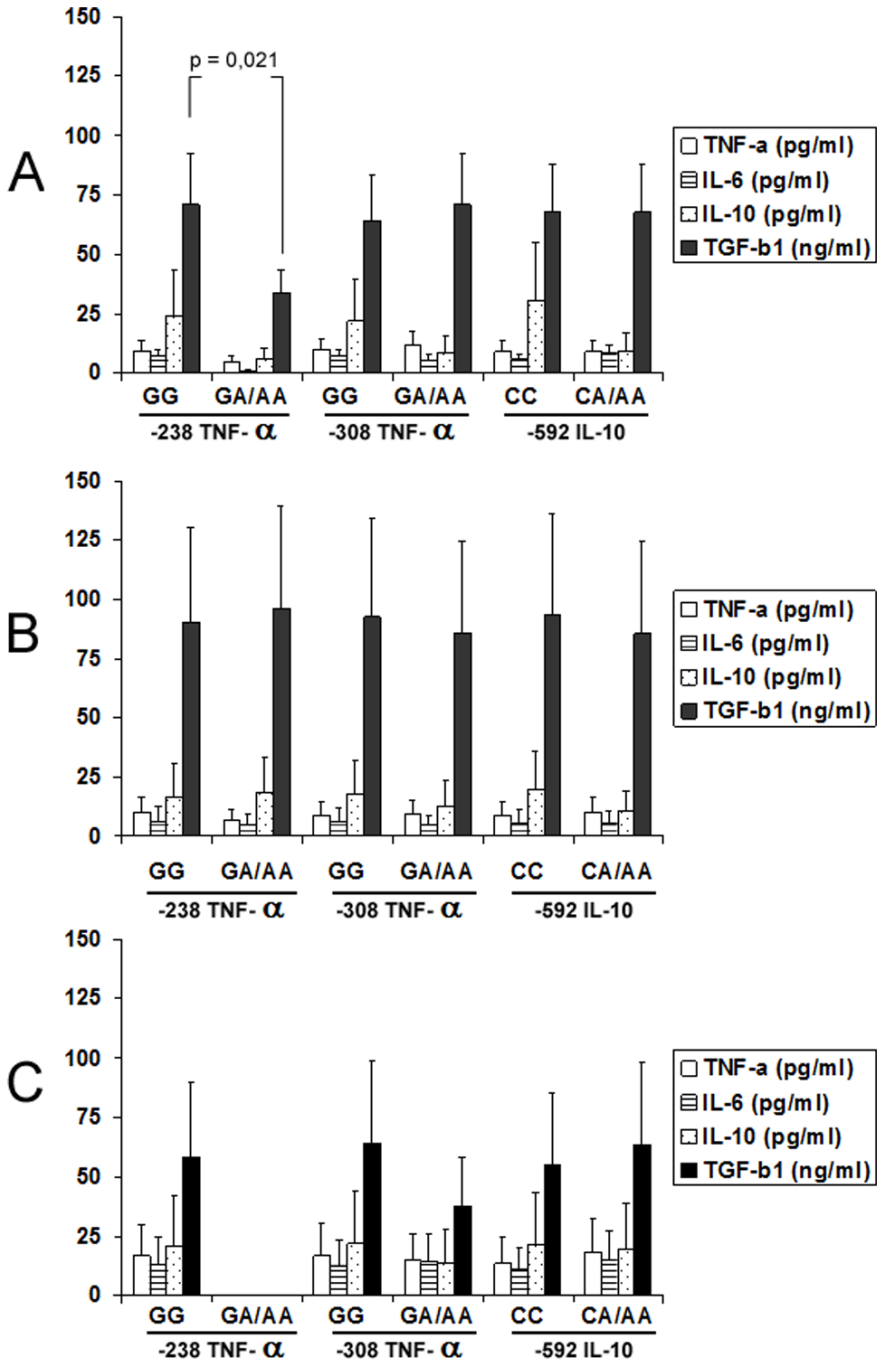
Serum concentrations of tumor necrosis factor-α (TNF-α) (white bars), interleukins 6 (IL-6) (lined bars) and 10 (IL-10) (dotted bars) and transforming growth factor beta 1 (TGF-β1) (black bars) in healthy controls (A) and in HIV-HCV coinfected patients with chronic hepatits (B) (n = 50) or liver cirrhosis (C) in function of the polymorphisms of -238 and -308 TNF-α and -592 IL-10 promotor genes.

Serum concentrations of TNF-α, IL-6, IL-10 and TGF-β1 were measured, separately, in healthy controls and in patients with HIV-HCV coinfection, distributed in two groups, those without and those with liver cirrhosis. In healthy controls, increased serum concentrations of TGF-β1 were detected in individuals with genotype GG at -238 position of the TNF-α promotor gene with reference to those with genotype GA or AA. The rest of comparison showed no significant differences among individuals with the different genotypes, both in healthy controls and in HIV-HCV coinfected patients (Figure 1).

Due to the relation between polymorphism at -238 position of the TNF-α promotor gene and serum concentration of TGF-β1, stimulation experiments were performed. Ten healthy controls with the genotype GG at -238 position of the TNF-α promotor gene and ten with the genotype GA were selected. No significant difference was detected when compared intracellular expression of TNF-α, IL-6, IL-10 and TGF-β1 in peripheral blood monocytes from both groups. LPS-stimulated secretion of these cytokines was analyzed in the culture supernatants. Peripheral blood mononuclear cells from healthy controls with the genotype GG at -238 position of the TNF-α promotor gene synthesize significantly higher concentrations of TGF-β1 than those of those with the genotype GA. Concentrations of TNF-α, IL-6 and IL-10 were similar in the culture supernatants from patients with genotype GG and GA (Table 4).

**Table 4:** Intracellular expression of tumor necrosis factor alpha, interleukin 6, transforming growth factor beta1 and interleukin 10 by monocytes from healthy individuals distributed in function of the -238 promotor gene polymorphism.

	GG polymorphism (n = 10)	GA polymorphism (n = 10)	P
**Intracelular expression of cytokines (% of monocytes)**			
TNF-α	1 (0 – 1)	1 (0 – 8)	1,000
IL-6	11 (4 – 14)	3 (3 – 14)	0,240
TGF-β1	99 (98 – 100)	99 (99 – 100)	0,240
IL-10	9 (6 – 16)	6 (2 – 21)	0,606
**Basal and LPS-stimulated secretion of TNF-α(pg/ml)**			
Basal	174 (131 – 259)	237 (227 – 324)	0,060
After LPS	648 (472 – 926)	871 (695 – 1549)	0,112
**Basal and LPS-stimulated secretion of IL-6 (pg/ml)**			
Basal	67 (12 – 103)	81 (41 – 140)	0,699
After LPS	2201 (1737 – 2977)	2180 (1568 – 2751)	0,797
**Basal and LPS-stimulated secretion of TGF-β1 (pg/ml)**			
Basal	32678 (24731 – 37118)	26456 (18664 – 31829)	0,364
After LPS	39553 (33129 – 43763)	28042 (21499–31576)	0,019
**Basal and LPS-stimulated secretion of IL-10 (pg/ml)**			
Basal	58 (0 – 66)	42 (0 – 64)	0,699
After LPS	72 (58 – 82)	78 (75 – 100)	0,112

Basal and LPS-stimulated concentration of tumor necrosis factor alpha, interleukin 6, transforming growth factor beta1 and interleukin 10 on supernatants of monocyte cultures. Abbreviations: TNF-α tumor necrosis factor alpha. IL-6, interleukin 6. TGF-β1, transforming growth factor beta 1. IL-10, interleukin 10. LPS, lipopolysaccharide.

### Multivariate analysis of parameters associated with cirrhosis

We evaluated, using a linear regression analysis, those factors independently associated to cirrhosis. The evaluated parameters were those whose differences among patients with chronic hepatitis and those with liver cirrosis were lower than 0,1 (age, alcohol abuse, CD4 T cell count at inclusion, TNF -238 and IL-10 -592 polymorphism) in the bivariate analysis and those other with possible clinical significance (CD4 T cell count at diagnosis of HIV infection) (Table 5). Continuous variables were categorized in function of their median values. In the resultant model, the polymorphism of TNF-α at -238 was the only factor associated with cirrhosis. Kaplan-Meier curves in function of the TNF-α -238 polymorphism are shown in Figure 1.

**Table 5:** Multivariant analysis of factors associated with the development of liver cirrhosis in HIV-HCV coinfected patients.

	HIV-HCV coinfected patients with chronic hepatitis (n = 50)	HIV-HCV coinfected patients with liver cirrhosis (n = 41)	Univariant p	Standardized coeficient beta (IC 95%) Multivariant p
**General characteristics**				
Age > 45 years (n, %)	25 (50)	28 (68)	0,121	
Alcohol ingestion (> 50 g/day) (n, %)	23 (49)	32 (78)	0,004	
**HIV-related characteristics**				
CD4+ T cell count at HIV diagnosis > 170/mm3 (n, %)	29 (59)	14 (37)	0,004	
CD4+ T cell count at inclusion > 343/mm3 (n, %)	29 (58)	16 (39)	0,111	
**Single nucleotyde polymorphism**				
TNF rs361525 (- 232) GG (n, %)	36 (77)	38 (97)	0,025	0,295
				(0,112–0,736)
				P = 0,008
IL10 rs1800872 (- 592) CC (n, %)	29 (59)	14 (36)	0,040	

When the patients diagnosed by liver biopsy were exclusively considered, we also detect that the polymorphism of TNF-α at -238 was the only factor associated with cirrhosis (standardized coeficient beta (IC 95%), 0,395 (0,156–0,534), p = 0,001).

## Discussion

Several factors, including age, alcohol abuse, duration of the HCV infection or the presence of immunodepression, influence the natural history of HCV-induced chronic hepatitis to cirrhosis [1], [2], [29], [30], [31]. However, the contribution of genetic factors to this evolution has been rarely examinated. Moreover, the independent contribution of genetic modifications to the evolution to liver cirrhosis in HIV-HCV coinfected patients has not been practically studied, with the exception of the IL28 B polymorphism [36].

The present work has analyzed the influence of genetic parameters on the risk of cirrhosis in a series of HIV-HCV coinfected patients. Selected genetic markers were those influencing the expression or secretion of molecules implicated in innate or acquired immunity, such as the TNF-α and IL-10. In this cohort of Caucasian patients, we found that TNF-α -238 polymorphism was involved in the risk of liver cirrhosis. Our data also indicated that differences in disease outcome among HIV-infected patients with established chronic HCV infections could have more to do with immunogenetic factors than with other factors previously accepted (age, gender, alcohol consumption, immunodepression), although several of them were associated with cirrhosis in the univariant analysis. This association persisted when only those patients with histologic diagnosis of chronic hepatitis or liver cirrhosis were considered.

The A allele at -238 position of the TNF-α promotor gene has been associated with a more strict virological control in HIV-infected patients [37]. Moreover, several studies in HCV-monoinfected patients have reported that the A allele at -238 position of the TNF-α promotor gene was associated with development of chronic active hepatitis C, advanced fibrosis progression or higher risk of cirrhosis [16], [17], although others had no demonstrated any association [18]. Our study in HIV-HCV coinfected patients has demonstrated precisely the opposite finding: those patients with a genotype GG shows an increased association with cirrhosis. The discrepancy could be explained by the consideration in our study, and not in the others, of the several factors which can be implicated in the evolution (age, sex, alcohol abuse), those related with the immunodepression (CD4 T cell count at diagnosis or at inclusion), as well as the evaluation of the independent influence of each one in a multivariant analysis.

The A allele at -238 position of TNF-α promoter gene has been associated with a more intense inflammatory activity [22]; however, data about its influence on parameters associated to fibrosis are lacking. After TNF-α activation, Kupffer cells secrete TGF-β1 [9], the more important fibrogenic molecule [38]. Levels of serum TGF-β1 have been correlated with the degree of liver fibrosis in patients with HCV-induced chronic hepatitis [39].

In patients with HCV-induced chronic hepatitis or cirrhosis an increase of several of these cytokines have been demonstrated, being related with the liver inflammatory activity, with the immune system activation and/or with the increased intestinal permeability [5], [10], [11], [12], [18]. Consequently, serum levels of these cytokines are not a reliable parameter of the innate ability of inflammatory/immune cells to secrete them in patients with hepatopathies. Thus, it is important stressed that in our study the association among -238 TNF-α polymorphism and serum concentration of TGF-β1 was observed in healthy individuals, in which others factors influencing TGF-β1 secretion are presumibly not involved. Our study has demonstrated that LPS-stimulated monocytes from healthy controls with the genotype GG synthesize significantly higher concentrations of TGF-β1 than those with genotype non-GG and that the serum concentration of the TGF-β1 is increased in healthy controls with the genotype GG in comparison with those with genotypes GA or AA. The other proinflammatory (TNF-α, IL-6) and anti-inflammatory (IL-10) molecules analyzed showed no significant different levels in healthy controls.

This is the first article in which the constitutive ability of monocytes to secrete fibrogenic factors in function of the TNF promoter gene polymorphism has been assayed. Three explanations can be proposed: 1) Linkage disequilibrium with TNF-α genes is not probable because TNF-α is codified by chromosome 6 [40] and TGF-β1 by chromosome 19 [41]. 2) Presence of polymorphisms of the TGF-β1 gene with influence on the secretion of this cytokine. This topic has not been studied in the present work. 3) The gene encoding TNF-α is located within the human leukocyte antigen (HLA) class III region, a region which is positioned between the HLA class I and class II region. About 40% of the genes that are encoded within the HLA-region are involved in immune processes [40]. Owing to the location of the TNF-α gene within the HLA-region, the relation between HLA-haplotypes and TNF-α polymorphisms, including the influence on disease severity or chronic hepatitis evolution to cirrhosis, is possible. As a factor implicated in the evolution of inflammatory diseases, it can be hypothesized the influence of TNF-α polymorphism in a complex inflammatory and immune cascade inducing an increased ability to secrete TGF-β1. In each case, the more intense fibrogenic activity, detected in patients with genotype GG, could be influencing the collagen secretion by hepatic stellate cell, favoring the fibrosis progression in the liver.

**Figure 2:**
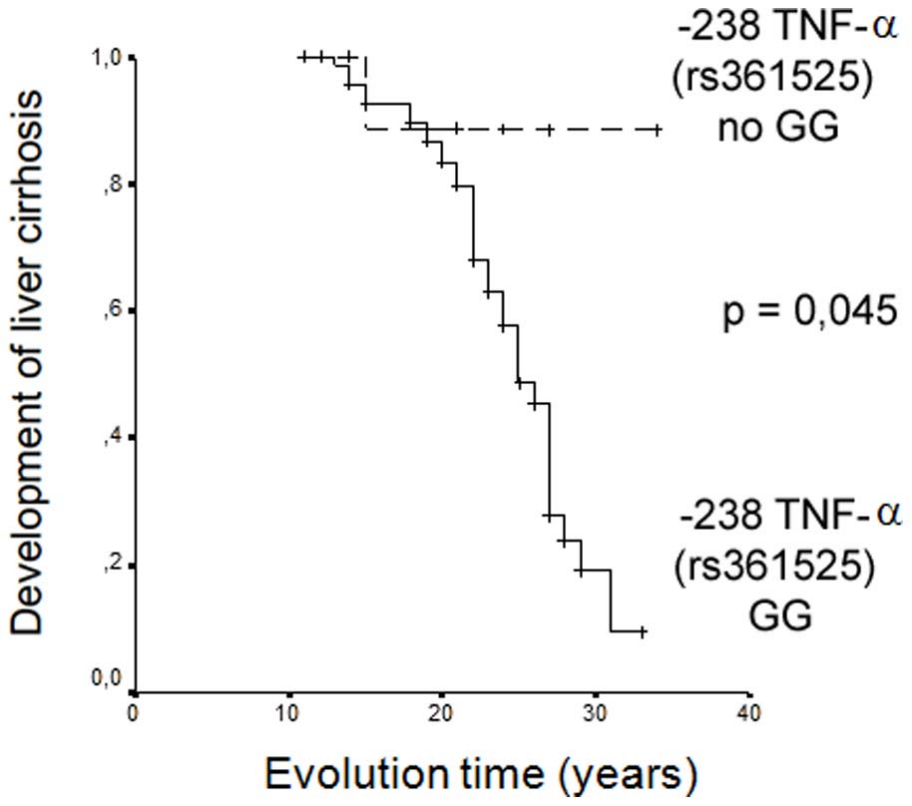
Kaplan Meier curves of the development of liver cirrosis in HIV-HCV patients in function of the polymorphism at -238 position of the TNF-α promoter gene.

The influence of the GG genotype at -238 position of TNF-α promoter gene was observed after a prolonged period: in fact, Kaplan Meier analysis demonstrated that curves of development of cirrhosis in function of the presence of genotypes GG or GA and AA at -238 position of TNF-α promoter gene begin to be clearly different after 20 years of evolution (Figure 2).

IL-10 is a cytokine which down-regulates the inflammatory response and modulate the liver fibrogenesis [42]. The polymorphism at -592 position of the IL-10 promoter gene has been associated with more accelerated progression of HIV infection [43] and with persistent HCV infection [28]. Data about influence on liver fibrosis progression are controversial [24], [25], [26], [27]. In our series, the genotype CA at -592 position of the IL-10 promoter gene was associated with a near significant higher frequency of liver cirrhosis in the bivariant analysis. This haplotype has been correlated with a decreased synthesis of IL-10 [23], suggesting that a lower anti-inflammatory activity could be implicated in the progression of liver fibrosis. However, serum concentrations of IL-10 were similar in healthy controls with the different IL-10 genotypes in our study. Moreover, the polymorphism at -592 position of the IL-10 promoter gene was not associated with cirrhosis in the multivariant analysis.

Several characteristics support the validity of our results: a) No significant difference existed between the distribution of TNF-α and IL-10 genotypes in the healthy control and HIV-HCV groups. b) Populations of Spanish healthy controls and HIV- or HCV- infected patients shows similar frequency of the genotypes tested [37], [44]. c) Data were similar when only those patients diagnosed of chronic hepatitis or cirrhosis by liver biopsy were considered**.** d) The evolution time from the infection until the moment of the inclusion was similar in HIV-HCV patients with chronic hepatitis and those with liver cirrhosis, excluding the possibility that cirrhosis developed exclusively as a consequence of a higher time with the infection. e) Patients were carefully evaluated for every one of the different known factors influencing the evolution to cirrhosis (age, sex, ethanol abuse, CD4+ T cell count at diagnosis and at inclusion, antirretroviral therapy or undetectable HIV viral load).

In conclusion, a GG genotype at -238 position in the TNF-α promoter gene influences the risk of liver cirrhosis in HIV-HCV infected patients, even more than those classically accepted factors influencing the progression of liver fibrosis. The implications of the influence of this polymorphism include the need of a more strict vigilance and counseling about other risk factors in them.
